# Comparative secretome analysis suggests low plant cell wall degrading capacity in *Frankia *symbionts

**DOI:** 10.1186/1471-2164-9-47

**Published:** 2008-01-28

**Authors:** Juliana E Mastronunzio, Louis S Tisa, Philippe Normand, David R Benson

**Affiliations:** 1 Department of Molecular and Cell Biology, U-3125, University of Connecticut, Storrs, CT, USA; 2 Department of Microbiology, University of New Hampshire, Durham, NH, USA; 3 Ecologie Microbienne UMR CNRS 5557, Université Lyon I, Villeurbanne 69622 cedex, France

## Abstract

**Background:**

*Frankia *sp. strains, the nitrogen-fixing facultative endosymbionts of actinorhizal plants, have long been proposed to secrete hydrolytic enzymes such as cellulases, pectinases, and proteases that may contribute to plant root penetration and formation of symbiotic root nodules. These or other secreted proteins might logically be involved in the as yet unknown molecular interactions between *Frankia *and their host plants. We compared the genome-based secretomes of three *Frankia *strains representing diverse host specificities. Signal peptide detection algorithms were used to predict the individual secretomes of each strain, and the set of secreted proteins shared among the strains, termed the core *Frankia *secretome. Proteins in the core secretome may be involved in the actinorhizal symbiosis.

**Results:**

The *Frankia *genomes have conserved Sec (general secretory) and Tat (twin arginine translocase) secretion systems. The potential secretome of each *Frankia *strain comprised 4–5% of the total proteome, a lower percentage than that found in the genomes of other actinobacteria, legume endosymbionts, and plant pathogens. Hydrolytic enzymes made up only a small fraction of the total number of predicted secreted proteins in each strain. Surprisingly, polysaccharide-degrading enzymes were few in number, especially in strain CcI3, with more esterolytic, lipolytic and proteolytic enzymes having signal peptides. A total of 161 orthologous proteins belong to the core *Frankia *secretome. Of these, 52 also lack homologs in closely related actinobacteria, and are termed "*Frankia-*specific." The genes encoding these conserved secreted proteins are often clustered near secretion machinery genes.

**Conclusion:**

The predicted secretomes of *Frankia *sp. are relatively small and include few hydrolases, which could reflect adaptation to a symbiotic lifestyle. There are no well-conserved secreted polysaccharide-degrading enzymes present in all three *Frankia *genomes, suggesting that plant cell wall polysaccharide degradation may not be crucial to root infection, or that this degradation varies among strains. We hypothesize that the relative lack of secreted polysaccharide-degrading enzymes in *Frankia *reflects a strategy used by these bacteria to avoid eliciting host defense responses. The esterases, lipases, and proteases found in the core *Frankia *secretome might facilitate hyphal penetration through the cell wall, release carbon sources, or modify chemical signals. The core secretome also includes extracellular solute-binding proteins and *Frankia*-specific hypothetical proteins that may enable the actinorhizal symbiosis.

## Background

A variety of plants, called actinorhizal plants, form N_2_-fixing root nodules when in symbiosis with actinobacteria of the genus *Frankia*. The molecular interactions governing this symbiosis, such as those involved in signaling between the bacteria and plant, or in penetrating the plant cell wall, are not well characterized, primarily because of the lack of genetic tools for generating *Frankia *mutants, but also because frankiae grow very slowly [1]. The genome sequences of three *Frankia *strains have recently become available, representing groups of frankiae having different host specificities. *Frankia *sp. strain HFPCcI3 (CcI3) is a narrow host range *Casuarina *isolate, *F. alni *strain ACN14a (ACN) is a more cosmopolitan *Alnus *isolate, and *Frankia *sp. strain EaN1pec (EAN) is a broad host range strain, isolated from *Elaeagnus *[2]. Although the strains are closely related (97.8–98.9% identity of 16S rRNA genes), their genomes range in size from 5.4 Mbp for CcI3, to 7.5 Mbp for ACN, to 9.0 Mbp for EAN [2]. In addition to the differences in host range and genome size, strains CcI3 and ACN also differ from strain EAN in carbon source usage in culture and in the manner of root infection. EAN can transport and grow on sugars such as fructose, sorbitol, or mannitol using a phosphotransferase system, while CcI3 grows best on pyruvate, and ACN on propionate or acetate [1]. Only EAN can penetrate plant roots intercellularly and via root hair infection, while CcI3 and ACN enter root cells solely through the latter method [1].

An examination of the frankial genomes recently revealed that *Frankia *strains lack the common *nod *genes involved in synthesizing signals in legume endosymbionts (*Rhizobium *and related genera), so an alternative system of chemical signaling between the plant and bacterium to form the root nodule must exist. It is likely that secreted proteins play a role in host-microbe interactions because of the intimate contact between plant and bacterium that occurs within the plant cell. During infection, *Frankia *hyphae within root cells are encapsulated by plant cell wall material, deposited as the organism penetrates from cell to cell, and consisting of cellulose, pectin, and xylan [3,4]. Cellulases, pectinases, and proteases have been reported in the culture medium of various *Frankia *strains, and have been proposed to participate in root infection [5-9]. Such proposals are logical since many members of the actinobacteria are nutrient scavengers that secrete enzymes which break down biopolymers and other compounds [10,11]. *Acidothermus cellulolyticus*, the closest known relative of *Frankia*, also in the suborder *Frankineae*, is noted for its cellulolytic ability. More distantly related *Streptomyces *species secrete chitinases, xylanases, cellulases and proteases into the soil environment [10]. Bacterial and fungal plant pathogens are also known to secrete many hydrolytic enzymes [12,13]. In contrast, legume endosymbionts (*Rhizobium *and other α-proteobacteria) do not appear to rely on hydrolases during nodulation, though this topic is still a matter of debate [14]. Indeed, it may be counterintuitive for a plant mutualist to secrete enzymes that degrade host tissue, since pectin cell wall fragments have been shown to act as endogenous elicitors of plant defense responses [15]. On the other hand, polysaccharolytic activity reported in *Frankia *strains, with no observed utilization of breakdown products (e.g. glucose), implies an additional function for these enzymes.

Protein secretion in Gram-positive bacteria is mediated mainly by the general secretory (Sec) and twin arginine translocation (Tat) pathways, and to a lesser extent by ABC (ATP-binding cassette) transporters and other minor pathways such as the ESAT-6 system in mycobacteria [16,17]. Proteins are targeted to both the Sec and Tat secretion systems by means of an N-terminal signal peptide. Both types of signal peptides contain a positively charged N-region, followed by a hydrophobic H-region, and ending with a cleavage site, recognized by signal peptidase I in the Sec system [18]. Tat signal peptides have a longer N-region containing a twin-arginine consensus motif, a less hydrophobic H-region, and "Sec avoidance" residues near the cleavage site [19]. In this work, we used SignalP 3.0 and TATFIND 1.4 to predict the individual secretomes (sets of secreted proteins) of each *Frankia *strain, as well as the secretome shared by all three strains, which we termed the core *Frankia *secretome [20-22]. The *Frankia *secretomes and secretion machinery genes were compared to those of related actinobacteria and other plant-associated bacteria. We reasoned that secreted proteins needed for symbiosis-specific processes are likely to be conserved in the core *Frankia *secretome, and may lack homologs in closely related actinobacteria. This comparative genomics-based secretome analysis defines proteins potentially involved in the actinorhizal symbiosis.

## Results and Discussion

### Secretion systems

**Figure 1:**
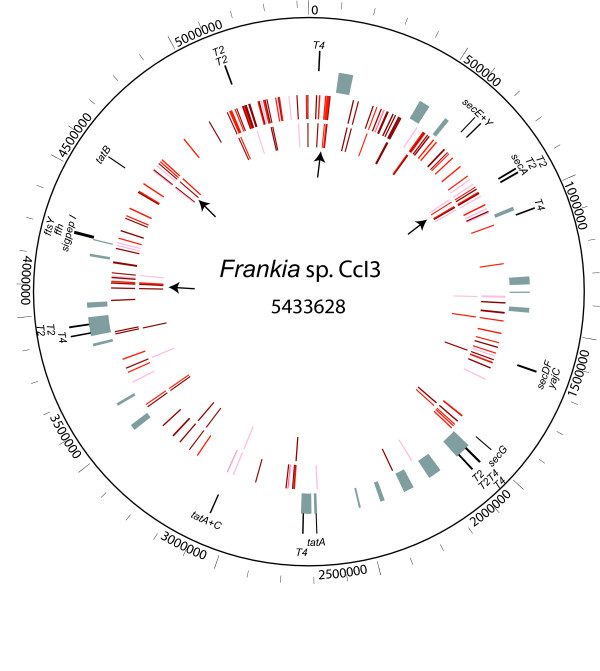
**Genome map of strain CcI3**. Circles, from the outside, show (1) the coordinates in bp, beginning at 0 = *dnaA*; (2) genes encoding components of the Sec and Tat pathways, and homologs to Type II (T2) and Type IV (T4) secretion proteins; (3) putative prophage regions, consisting of hypothetical proteins bordered by phage integrases; (4) ORFs in the core *Frankia *secretome; (5) ORFs in the core *Frankia*-specific secretome. Colors indicate the number of strains in which a signal peptide was predicted: three (dark red), CcI3 and one other strain (medium red), or only ACN and EAN (light red). Arrows indicate clusters of conserved secreted proteins.

Before predicting the *Frankia *secretomes, the genomes were scanned for the presence of secretion systems found in other actinobacteria. Genes encoding conserved secretion machinery-related proteins were identified based on the COGs (Clusters of Orthologous Groups) annotations, and are listed in Additional File 1. All genes necessary for the Sec translocation apparatus are present in each *Frankia *strain. These encode proteins forming the main membrane channel-forming complex, SecYEG, the cytosolic ATPase SecA, the auxiliary proteins SecD, SecF, and YajC, and the chaperones Ffh and FtsY. As in other Gram-positive bacteria, *secB *is not present [23]. The most closely related homologs to each of these genes are in other actinobacteria. The order and composition of genes surrounding the Sec machinery genes are similar or identical to those in the close actinobacterial relatives *Acidothermus cellulolyticus *and *Kineococcus radiotolerans*, and in the more distantly related *Streptomyces avermitilis *(data not shown). The locations of these genes in the CcI3 genome are depicted in the circular map in Figure 1 (second circle in), along with areas of putative prophage integration (third circle from outside). Genes encoding the twin-arginine translocase (Tat) system, *tatA, tatB*, and *tatC*, are also present in the three genomes. In *Frankia *and in the majority of other sequenced actinobacterial genomes, *tatC *is present in a single copy, with a *tatA *directly preceding it, while *tatB *is located separately from these genes (Figure 1). Each genome has at least one additional copy of *tatA*.

Eight genes are annotated as encoding components of a Type II secretion (T2S) system. The closest homologs to these T2S genes are found in other actinobacterial genomes. Gram-negative pathogens secrete toxins via T2S; proteins are translocated to the periplasm by the Sec or Tat system, and then across the outer membrane by the Type II complex [24]. The T2S proteins in *Frankia *are also similar to the Flp pilus assembly proteins such as the ATPase CpaF, TadA, or TadC. The *tad *(tight adherence) locus is widespread in bacteria and is involved in adhesion or secretion [25]. The T2S/Flp pilus/Tad homologs in the *Frankia *genomes occur in operons consisting of two or three genes. There are four of these operons in both the CcI3 and EAN genomes, but only two in the ACN genome. The two operons that are not shared with ACN (Additional File 1) coincide with putative prophages in CcI3 (Figure 1).

The CcI3 and EAN genomes have genes annotated as encoding VirB4 and VirD4 components of the Type IV secretion (T4S) system. Type IV secretory pathways transport macromolecules (proteins or DNA) across the cell envelope in Gram-negative and Gram-positive bacteria. VirB4 and VirD4, located in the cytoplasmic membrane, bind ATP and provide the energy for translocation [26]. The predicted T4S components have low sequence similarity to known T4S proteins, and instead resemble integral membrane proteins with ATP-binding domains. Only one putative VirB4 homolog is conserved among all three strains (CcI3 gi|86738757) and has the highest similarity to an ATP-binding protein from *Streptomyces *species. CcI3 and EAN each have VirB4- and VirD4-related proteins that are not found in the ACN genome, and most of these are located near phage integrase or excisionase genes.

Based on the observation that genes encoding both the Sec and Tat secretion machinery in *Frankia *are not only present, but located in conserved regions (in the case of *tatA/tatC*) in other actinobacterial genomes, it is likely that both of these systems are functional in *Frankia*. In contrast, it is less clear whether the proteins annotated as "Type II secretion" and "VirB4/D4" homologs constitute significant export pathways in *Frankia*, as very low sequence similarity is seen between the frankial genes and the known T2S and T4S proteins. These putative secretion machinery genes are not well conserved across the three strains, and appear to be associated with prophage regions in most cases.

### Individual *Frankia *secretomes

**Figure 2:**
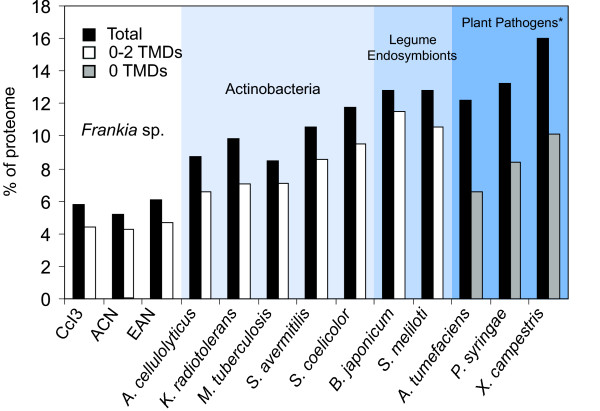
**Comparative secretome sizes**. The secretome of each organism is shown as the percentage of coding sequences in the proteome with signal peptides predicted by both SignalP methods (NN & HMM), and containing 0–2 transmembrane domains (TMDs). The genomes of *Frankia *strains, actinobacteria and legume endosymbionts were analyzed with SignalP 3.0 and TMHMM as described in the Methods. *Data for plant pathogens (analyzed with SignalP, but only reporting proteins with zero TMDs) were adapted from Preston *et al*., 2005.

The complete CDS (coding sequence) translations from each frankial genome were analyzed with SignalP 3.0, and then scanned for transmembrane domains with TMHMM v. 2.0 [22,27]. The predicted secretomes for individual strains consist of proteins with signal peptides predicted by both SignalP methods, neural networks (NN) and hidden Markov models (HMM), and containing between zero and two transmembrane (TM) domains. These totals, shown in Table 1, Columns 1–3 (numbers outside parentheses), have been used to estimate the number of secreted proteins in other genome-based secretome studies [28]. The secretomes comprise similar percentages of the proteomes of each strain: 4.4% (197 of 4499 CDS) in CcI3, 4.1% (279 of 6711 CDS) in ACN, and 4.8% (346 of 7191 CDS) in EAN (Table 1, Figure 2). Since the annotation for *F. alni *strain ACN was performed by MaGe [29] rather than the Joint Genome Institute [30], differences in the annotation of start codons were observed, which may account for differences in signal peptide predictions (see Methods).

**Table 1:** Signal peptide-containing proteins in individual and core secretomes. Only sequences containing 0–2 transmembrane domains (TMDs) as predicted by TMHMM are shown.

Function	CcI3*	ACN*	EAN*	Core secretome^†^	Core *Frankia*-specific^‡^
Cell Wall/Growth	14 (4)	13 (6)	15 (8)	17	6
Hydrolase	10 (9)	17 (9)	21 (6)	12	4
Metabolism	42 (99)	55 (159)	56 (163)	59	7
Regulation	10 (15)	13 (19)	11 (20)	10	2
Solute-binding	18 (1)	28 (8)	62 (12)	14	4
Hypothetical	103 (110)	153 (177)	181 (175)	49	29

Total	197 (238)	279 (378)	346 (384)	161	52

* Numbers outside parentheses are predicted to contain signal peptides by both SignalP methods (NN and HMM); numbers inside parentheses are additional proteins predicted to contain a signal peptide by only one method.

^† ^Orthologous proteins present in all strains with a signal peptide predicted in two or three strains

^‡ ^*Frankia*-specific: when CcI3 sequences from the core secretome list were BLAST searched against four closest actinobacterial relatives, no hits retrieved at an E value cutoff of 10^-20^

Table 1 categorizes the secretome proteins based on annotations from the NCBI database, and a complete list of potentially secreted proteins can be found in Additional File 2. "Cell wall/Growth" includes peptidoglycan-binding proteins and transglycosylases. "Hydrolases" are also listed in Table 2, and are addressed in more detail in the discussion below. "Metabolism," the broadest group, encompasses dehydrogenases, transferases, phosphatases, and enzymes with broadly defined functions. "Regulation" usually refers to sensor histidine kinases, while "solute-binding" refers to extracellular binding of molecules presumably for transport into the cell. Hypothetical proteins accounted for roughly half of the proteins in the individual secretomes.

**Table 2:** Hydrolases with signal peptides in one or more strains

	**Gene present in three strains**	CcI3 gi	ACN gi	EAN gi
Polysaccharolytic	Glycoside hydrolase, family 16	86742522	111222085	158319021^†^
Esterolytic/Lipolytic	Patatin	86739488	111220771^‡^,111222061^†^	158317574, 158312853
	Poly(3-hydroxybutyrate) depolymerase-like*	86738795	111219601^†^	158312237, 158318953
	Poly(3-hydroxybutyrate) depolymerase-like*	86743135	111224988	158313427, 158312582
	Poly(3-hydroxybutyrate) depolymerase-like*	86739947	111221400^†^	158314094
	Poly(3-hydroxybutyrate) depolymerase-like*	86742257	111225134^†^	158313019^†^
	Putative esterase	86741433	111223634^†^, 111224716^†^	158313383, 158315460
	Secretory lipase*	86738753	111219534^‡^, 111224342	158311883^†^
Proteolytic	Metalloprotease-like protein	86741105	111223246^†^	158315133
	Peptidase M16-like	86742474	111225385^†^	158312786^†^
	Peptidase M22, glycoprotease	86739340^†^	111220592	158317749^†^
	Peptidase M23B	86742263^†^	111225140	158313012
	Peptidase S1 and S6, chymotrypsin/Hap	86738792	111219598	158318956^†^
	Peptidase S1 and S6, chymotrypsin/Hap	86740691	111223367^‡^	158318071
	Peptidase S8 and S53 subtilisin kexin sedolisin	86742624	111225604	158312643
	Peptidase S15	86742910^†^	111224424^†^	158315881
	Peptidase S16 lon domain protein	86742463	111225373	158312797
	Putative peptidase domain	86742587^†^	111225562	158312677
Hypothetical	Alpha/beta hydrolase fold	86739338	111220590^†^	158317752
	Alpha/beta hydrolase fold	86742456	111225365^†^	158312806^†^
	Alpha/beta hydrolase fold	86741553	111223803	158313859^†^
	Hypothetical protein; putative glycosidase*	86742141	111222591	158317255
	Hypothetical protein; putative glycosidase*	86739771	111221236	158312651^†^
	Hypothetical protein; putative lipase	86742918	111225845	158318206
	Hypothetical protein; putative GDSL lipase	86743055^†^	111226008^‡^	158312060
	Hypothetical protein; putative xylanase	86741387	111220334	158314619
				
	**Gene present in two strains**			
Polysaccharolytic	Cellulase*	x	111224344^†^, 111224345^†^	158313493^†^, 158318914
	Glycoside hydrolase, family 3, N-terminal	x	111220352	158317032
	Glucan endo-1,3-beta-D-glucosidase	x	111221161	158318718
Esterolytic/Lipolytic	Esterase	x	111221893	158313421^†^
	Feruloyl esterase*	x	111221879	158315070
	Lipase class 2	86739361^†^	x	158312565
	Putative carboxylesterase/lipase	x	111221691	158315258^†^
	Putative lipase	x	111223894, 111223109	158314118^†^
Proteolytic	Peptidase S8 and S53, subtilisin	x	111224419	158314198
	Putative protease	86741028	x	158315902
				
	**Gene present in one strain**			
Polysaccharolytic	Arabinogalactan endo-1,4-beta-galactosidase	x	x	158314089
	Glycoside hydrolase, family 26	86741945	x	x
	Glycoside hydrolase, family 32	x	x	158318776
	Levanase	x	x	158318763
	Putative glycosidase	x	111223066	x
	Putative xylanase	x	111222228	x
Esterolytic/Lipolytic	Lipolytic enzyme, G-D-S-L	x	111222700	x
	Phospholipase A2	x	x	158315136
Hypothetical	Alpha/beta hydrolase fold	86741921	x	x

**Figure 3:**
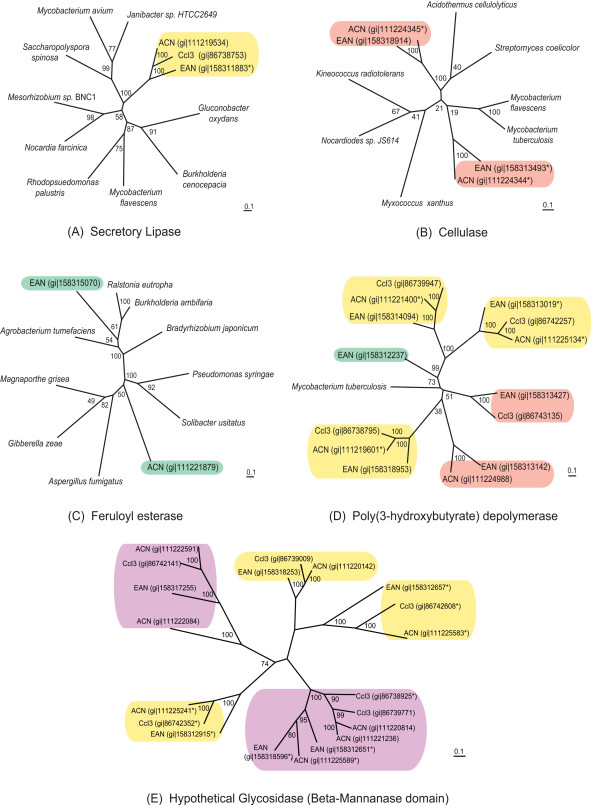
**Phylogenetic trees of secreted hydrolytic enzyme genes**. Various patterns of gene retention and acquisition are observed in *Frankia *hydrolase genes, including (A) conservation of an ancestral gene; (B) gene loss in one strain; (C) horizontal acquisition; (D) and (E) gene family expansion (see text). Trees were generated using PhyML (maximum likelihood method). *not predicted to contain a signal peptide by SignalP or TATFIND 1.4.

* Phylogenetic trees shown in Figure 3. For hypothetical glycosidases, Figure 3(E) shows addtional homologs not listed here.

^† ^Not predicted to contain a signal peptide by SignalP or TATFIND 1.4

^‡ ^Signal peptide predicted after manual inspection/re-annotation of start site region (see Methods)

Table 1 includes some proteins also predicted to contain Tat signal peptides. In CcI3, TATFIND 1.4 predicted 30 Tat signal sequences, and 20 of these were also predicted to have Sec signal peptides. Likewise, 22 of 31 Tat signal sequences in ACN and 48 of 76 in EAN had signal peptides predicted by both TATFIND and SignalP. The results of the TATFIND analysis, including SignalP predictions and Tat signal peptide sequences, can be found in Additional File 3.

### Comparison with secretomes from other bacteria

We analyzed seven other bacterial genomes with SignalP and TMHMM: five actinobacterial relatives of *Frankia*, and two legume endosymbionts, *Bradyrhizobium japonicum *and *Sinorhizobium meliloti*. Figure 2 compares the secretomes of each of these, reported as a percentage of the total proteome. Data for three plant pathogen genomes, adapted from Preston *et al*. (2005), are also included [28]. The secretomes of the two closest relatives of *Frankia, Acidothermus cellulolyticus *and *Kineococcus radiotolerans*, comprise higher percentages of the total proteome of each species (6.6% and 7%). Roughly 30% of these secreted proteins are shared with those of *Frankia*, while another 30% are hypothetical proteins not found in *Frankia *(using an E value cutoff of 10^-20^). Notable differences include ten cellulose-degrading enzymes in *A. cellulolyticus *and 20 extracellular solute-binding proteins for transport of sugars in *K. radiotolerans*. The secretome of *Mycobacterium tuberculosis*, making up 7.1% of its proteome, is enriched in lipoproteins and *Mycobacterium*-specific surface proteins of the PE_PGRS family. The secretomes of the two *Streptomyces *species make up higher percentages of their proteomes (8.6% and 9.5%), roughly double the percentages seen in *Frankia*, and contain many potential secreted hydrolytic enzymes (100 in *S. avermitilis *and 134 in *S. coelicolor*). The legume symbionts and the plant pathogens analyzed have more than double the percentages of secreted proteins predicted in *Frankia *(Figure 2). Fewer secreted hydrolases are predicted in legume endosymbionts than in plant pathogens, but both secrete substantially more solute-binding proteins than are found in the *Frankia *genomes, suggesting a wider range of nutritional sources used. The comparatively small size of the *Frankia *secretomes may reflect the narrow scope of carbon sources utilized by *Frankia *strains, especially strains CcI3 and ACN, which have less than half of the solute-binding proteins found in EAN (Table 1, Additional File 2). The secretion of relatively few proteins could also affect its recognition as "friend or foe" by host plants. Unlike the extensive biopolymer-degrading capability observed in *Streptomyces*, it may be beneficial for *Frankia *to secrete few degrading enzymes in its mutualistic interactions with plants.

### Secretory hydrolases

A major objective of the current study was to screen the *Frankia *genomes for secreted hydrolytic enzymes, which have been hypothesized to play a role in nodulation. As seen in Table 1, the *Frankia *genomes have between 10–21 hydrolases with signal peptides predicted by both SignalP methods (NN and HMM), and an additional 6–9 predicted by only one SignalP method. Table 2 lists all hydrolytic enzymes identified in at least one *Frankia *strain with signal peptides predicted by at least one SignalP method (NN or HMM). Those marked with "‡" had signal peptides predicted only after manual inspection of the N-terminal alignments, and adjustment of the start sites to match those of the other two strains (see Methods). Sequences listed in the same row retrieved one another in a BLAST search using a permissive E value cutoff of 10^-5^. An "x" in Table 2 indicates the absence of any hits to other frankial sequences at this E value. In many cases, sequences with signal peptides predicted in one strain lack signal peptides in the corresponding proteins in other strains (denoted by †). This may be due to ancestral N-terminal sequences having diverged significantly in each lineage. Alternatively, the similarly annotated proteins within a row may actually only share a conserved domain, but belong to different families. If this is the case, the presence or absence of a signal peptide in one or more strains may indicate distinct ancestral origin, with only superficial (or convergent) similarity to other sequences retrieved in a BLAST search.

Of the secretory hydrolases, lipolytic and esterolytic enzymes had the most signal peptides predicted (Table 2). Lipases and esterases secreted by frankial strains could depolymerize components of plant cell walls, or could modify lipid-based signaling molecules generated by plants in response to stress or pathogen attack [31]. There have been no direct reports of lipase activity in *Frankia *strains, though whole cell esterase zymograms have been used to distinguish between isolates, and the addition of long chain fatty acids to culture media enhances growth of some strains [32,33]. Lipase activity may be conferred by a conserved secretory lipase (discussed below) or by the putative lipases, one of which (gi|86743055 in CcI3) is similar to a GDSL lipase from *Streptomyces rimosus *that has demonstrated lipolytic activity [34]. Another possible lipolytic enzyme found in the three strains is annotated as patatin, which is a storage glycoprotein in potato tubers that can act as an acyl hydrolase. Patatin-like proteins are found in many bacterial species, and phospholipase A_2 _activity has been shown for the patatin homolog ExoU, an exotoxin secreted via Type III secretion by *P. aeruginosa *[35]. Esterolytic proteins in *Frankia *could potentially cleave ester linkages in complex plant polysaccharides, liberating compounds for use as carbon sources or signaling molecules. For example, GDSL lipases can act as acetylxylan esterases, and feruloyl esterases cleave ferulic acid from the sugar backbone of pectin [36,37]. The polyhydroxybutyrate (PHB) depolymerase-like proteins could degrade polyesters such as suberin, a polyester found in plant root cell walls that consists of lipids and phenolic compounds. The accumulation of suberin in cell walls around *Frankia-*infected cells has been observed in *Casuarina *nodules [38].

In contrast to lipases and esterases, polysaccharide-degrading enzymes with signal peptides are less prevalent (Table 2). Strain EAN, which has the largest genome and widest host range, has the most polysaccharolytic enzymes, followed closely by ACN, while CcI3 has the fewest, possessing only general "glycoside hydrolase" genes. Two putative cellulolytic proteins are found in strains ACN and EAN, with a signal peptide predicted only in one EAN sequence. CcI3 lacks both of these cellulase genes. EAN (EaN1pec) has a single pectate lyase gene (gi|158314134, not shown), which is absent from CcI3 and ACN. This protein was not predicted to contain a signal peptide, though it is most similar to a pectate lyase in *S. avermitilis *that has a predicted Tat signal peptide. Another putative pectin-degrading enzyme found only in strain EAN is an arabinogalactan endo-1,4-beta-galactosidase, which shows highest sequence similarity to this enzyme in *Burkholderia cenocepacia *(42% identity, E value of 10^-64^). *Frankia *strains share a group of hypothetical proteins ("putative glycosidases" in Table 2) with several features indicating that they may act as hydrolases: all share a beta-mannanase (ManB) domain, and several are similar to Glycoside Hydrolase Family 26 or have low hits to the (Trans)-Glycosidase protein superfamily (see Methods). These proteins may potentially interact with plant cell wall polysaccharides, or with glycoproteins or glycolipids on the frankial cell envelope. No clear polysaccharases have signal peptides predicted in all three strains.

Finally, a variety of proteolytic enzymes appear to be secreted by *Frankia*. Peptidases with clear housekeeping functions in growth and cell wall remodeling are omitted from Table 2. Extracellular peptidases have been detected in culture supernatants of *Frankia *strains [6,39]. Muller and Benoist [39] described a 1300 kDa proteinase complex, composed of 11 proteinase subunits, from both cell extracts and the extracellular concentrate of *Frankia *strain BR, a *Casuarina *isolate. Based on the cleavage specificity of the proteinase subunits, Benoist *et al*. suggested a role in degrading cell wall proteins such as extensins [7]. The two conserved signal peptide-containing serine proteases belonging to the S1/S6/Hap family may mediate attachment to host cell surfaces, as these are similar to the *Haemophilus influenzae *adhesin Hap, which binds to the extracellular matrix of human cells [40]. *Frankia *strains can use peptides and amino acids (and have branched-chain amino acid binding proteins with signal peptides) so the presence of proteases is not unexpected.

Phylogenetic trees were constructed to trace the origin of selected hydrolytic enzyme genes in the *Frankia *strains (asterisks in Table 2), and to provide insight on their relevance to the ecology of *Frankia*. Several patterns become apparent: first, conservation of a gene with a single copy in the three strains that is also present in actinobacterial ancestors. The secretory lipase gene, shown in Figure 3(A), is one of the few examples of this among hydrolases. A second pattern observed is the loss of a hydrolase gene in one or two strains. Losses have occurred most often in the genome of CcI3, which is the smallest of the three and has lost many other genes [2]. As shown in Figure 3(B), two cellulase genes are found in EAN and ACN and in closely related actinobacterial genomes, but have been lost from CcI3. Gene loss may account for the presence of other polysaccharases only in strains ACN and EAN. In contrast, several hydrolases appear to have been acquired separately by one or two *Frankia *strains. A feruloyl esterase gene is seen in both EAN and ACN, but there is minimal similarity between the sequences, and the tree in Figure 3(C) suggests that these did not originate from a common actinobacterial ancestor, but rather may have been acquired from distantly related soil microorganisms. The fourth notable pattern is that of gene family expansion within the *Frankia *lineage. The four to six poly-hydroxybutyrate (PHB) depolymerase-like gene families depicted in Figure 3(D) seem to have undergone duplication in a frankial ancestor. The *Frankia *sequences are more similar to each other than to any other sequences in the database; the outgroup shown belongs to the lipoprotein family LpqC (also in COG3509) of *Mycobacterium tuberculosis*. The expansion and retention of this group of genes points to *Frankia-*specific function, though interestingly, most of the sequences in strain ACN lack signal peptides, while these are predicted in the other two strains. Another gene family expansion is shown in Figure 3(E), which includes a total of 20 *Frankia *sequences, ten with signal peptides (see "putative glycosidases" in Table 2), and ten without. While non-*Frankia *sequences were retrieved in BLAST searches, these did not clearly associate with any *Frankia *sequences, and so were excluded from this unrooted tree.

In summary, esterases, lipases, and proteases are better represented across *Frankia *strains than polysaccharide-degrading enzymes. The presence of a few more polysaccharases in strain EAN may be a factor in EAN's ability to penetrate plant roots intercellularly via cracks in roots, or may reflect the wider carbon source usage of this strain during saprophytic growth [41]. The patterns of hydrolase gene retention, loss, acquisition, and duplication illustrated in Figure 3 emphasize the divergent life histories of individual strains, a topic addressed on a genome-wide scale by Normand *et al*. [2].

### The core *Frankia *secretome

Secreted proteins that are essential for the actinorhizal symbiosis are likely to be highly conserved in *Frankia *strains, and may lack homologs in closely related actinobacteria. To circumscribe this group of proteins, we first identified the core proteome, consisting of orthologous proteins in all three strains (top-scoring reciprocal BLAST hits, in the three bi-directional comparisons, using an E-value threshold of 10^-20^). At this E value, a total of 2080 sequences belonged to the core proteome. When the sequences of the core proteome (2080 sequences from each genome) were analyzed with SignalP, the signal peptide predictions for these orthologous proteins varied from strain to strain. It was thus necessary to define a limit for including a given protein in the core secretome. We defined the core secretome as proteins within the core proteome that had signal peptides predicted by at least one SignalP method (NN, HMM, or both) and zero to two TM domains, in at least two of the three strains. Of the initial 2080 proteins in the core proteome, 77 were predicted to contain signal peptides in all three strains, and 83 more had signal peptides in two strains; therefore, 161 proteins are estimated to encompass the core *Frankia *secretome (Table 1, column 4). Notably, only nine hydrolases belong to the core secretome, and these are mainly proteases, esterases, and lipases. The complete listing of proteins in the core secretome can be found in Additional File 4.

To narrow this set of proteins to those highly conserved within the *Frankia *lineage, the 161 sequences of the core secretome (from each genome) were BLAST searched against genomes of four actinobacterial species closely related to *Frankia*: *Acidothermus cellulolyticus, Kineococcus radiotolerans, Streptomyces avermitilis*, and *Nocardia farcinica*. A total of 52 sequences from the core secretome did not show significant alignment, at an E value cutoff of 10^-20^, to proteins of these close actinobacterial relatives. These proteins are termed *Frankia-*specific (see Table 1, column 5), and are listed in Table 3. About half of these sequences, especially the hypothetical proteins, did not retrieve any BLAST hits to actinobacterial proteins at a less stringent E value cutoff of 10^-5^. Since the sequence alignments have E values very close to zero across the three *Frankia *strains, it is probable that these proteins carry out novel functions, perhaps governing the interactions with host plants.

**Table 3:** The *Frankia*-specific core secretome

		CcI3 gi	ACN gi	EAN gi
**Cell wall/growth**	Lytic transglycosylase, catalytic	86742160^†^	111225025	158313119
	OmpA/MotB	86739073	111220228	158318192
	Peptidoglycan-binding domain 1	86741674	111224313	158314338
	Putative lipoprotein	86742138	111224994	158313137
	Septum formation initiator	86742601	111225576	158312664
	Transglycosylase-like	86742920	111225850	158318320
				
**Hydrolase**	Hypothetical protein; putative glycosidase	86742141	111222591	158317255
	Poly(3-hydroxybutyrate) depolymerase-like	86739947	111221400^†^	158314094
	Poly(3-hydroxybutyrate) depolymerase-like	86738795	111219601^†^	158318953
	Putative signal peptide; putative peptidase domain	86742587^†^	111225562	158312677
				
**Metabolism**	GCN5-related N-acetyltransferase	86743083	111226044^†^	158312032
	K+ transporting ATPase, KdpC subunit	86741971	111224671	158312459
	Lipopolysaccharide biosynthesis	86742615	111225592	158312648^†^
	Peptidyl-prolyl cis-trans isomerase, cyclophilin type	86740087	111221586	158316890
	Poly-gamma-glutamate biosynthesis protein	86738783	111219578	158318982
	Stage II sporulation E	86739884	111221318	158317049
	UvrD/REP helicase	86743179	111224714	158313386^†^
				
**Regulation**	Protein serine/threonine phosphatases	86743061	111226019	158312053
	Protein-tyrosine kinase	86742154	111225020	158313124^†^
				
**Solute-binding**	ABC-type branched-chain amino acid transport, periplasmic	86742125	111224977	158317330
	Extracellular ligand-binding receptor	86741514	111223750	158314112
	Extracellular solute-binding protein, family 1	86739179	111220428	158317895
	TRAP-type uncharacterized transport system, periplasmic	86739077	111220233	158318181
				
**Hypothetical**	Allergen V5/Tpx-1 related	86743037	111225986	158312084^†^
	Hypothetical protein Francci3_0037	86738760^†^	111219542	158319012
	Hypothetical protein Francci3_0040	86738763	111219548	158319008
	Hypothetical protein Francci3_0066	86738789	111219591^†^	158318965
	Hypothetical protein Francci3_0077	86738800	111219607	158318948
	Hypothetical protein Francci3_0189	86738909	111224111	158318981
	Hypothetical protein Francci3_0265	86738982	111220095	158318303^†^
	Hypothetical protein Francci3_0293	86739009	111220142	158318253
	Hypothetical protein Francci3_0740	86739453^†^	111220717	158317614
	Hypothetical protein Francci3_0760	86739473	111220756^†^	158317589
	Hypothetical protein Francci3_0772	86739485	111220768	158317577^†^
	Hypothetical protein Francci3_0777	86739490	111220773	158317572
	Hypothetical protein Francci3_0789	86739502	111220806	158317543
	Hypothetical protein Francci3_1158	86739866^†^	111221294	158317064
	Hypothetical protein Francci3_1658	86740363	111223957	158313918^†^
	Hypothetical protein Francci3_2782	86741470	111223676	158314336
	Hypothetical protein Francci3_3087	86741773	111224471	158313666
	Hypothetical protein Francci3_3155	86741841^†^	111224558	158313592
	Hypothetical protein Francci3_3200	86741886^†^	111224609	158313544
	Hypothetical protein Francci3_3489	86742171	111225037	158313108
	Hypothetical protein Francci3_3886	86742565^†^	111225535	158312696
	Hypothetical protein Francci3_3888	86742567	111225539	158312694^†^
	Hypothetical protein Francci3_4257	86742932	111225862	158312224^†^
	Hypothetical protein Francci3_4291	86742966	111225895^†^	158312188
	Hypothetical protein Francci3_4326	86743001^†^	111225939	158312147
	Hypothetical protein Francci3_4510	86743185	111226172	158319016
	Kelch repeat protein	86738890	111219845	158313864
	Protein of unknown function DUF37	86743221	111226215	158319056
	Protein of unknown function DUF459	86742591^†^	111225566	158312673

	^†^Not predicted to contain a signal peptide by SignalP or TATFIND 1.4			

Other proteins listed in Table 3 may have roles in the actinorhizal symbiosis. The proteins listed under "Metabolism" include a peptidylprolyl isomerase, which could assist in modifying the proline-rich proteins of the plant cell wall, or may ensure proper folding of secreted proteins. A peptidylprolyl isomerase (Mip) in *Legionella pneumophila*, in concert with a serine protease, facilitates entry into lung epithelial cells [42]. The lipopolysaccharide or poly-γ-glutamate biosynthesis proteins may alter cell surface characteristics, contributing to host recognition of the frankial cell envelope. The former has minimal similarity to other proteins in the non-redundant database, while the latter is similar (33% identity, E value of 10^-37^) to a capsule biosynthesis protein from *Bacillus thuringiensis*. Twelve solute-binding proteins are found in the core *Frankia *secreteome. The extracellular solute-binding protein (Family 1) has highly conserved sequence across *Frankia*, with the next best BLAST hits revealing low sequence similarity to sugar-binding proteins in *Mesorhizobium loti *and *Rhizobium leguminosarium *(E values of 10^-17^). TRAP-type transporters, akin to those in rhizobia, could transport dicarboxylic acids, known carbon sources of some *Frankia *strains. We propose that these conserved secreted proteins, especially those with little homology to other bacterial proteins, are likely candidates for symbiosis-related functions.

### Genome map

Mapping the gene locations of core secretome proteins on the CcI3 chromosome (Figure 1) revealed clustering of conserved secreted proteins, in some cases adjacent to secretion machinery genes. The outer two circles (after the scale) depict the secretion machinery genes, and putative prophage regions, respectively. The third circle in shows the genes encoding the proteins of the core secretome, while the fourth circle in depicts the genes for the proteins in the core *Frankia-*specific secretome. Colors indicate which strains have signal peptides predicted, as noted in the legend. None of the genes encoding core secretome proteins are located within prophage neighborhoods, and no horizontally transferred "secretion islands" are evident. Clustering of core secretome genes near the origin of replication is seen; this region of the chromosome also shows the most synteny among the three strains, and contains the *nif *genes required for nitrogen fixation [2]. Several clusters of core secretome genes are located close to secretion machinery genes (arrows, Figure 1). Beginning very close to the origin of replication, one putative secretory region of the *Frankia *CcI3 chromosome, from roughly 0.035 to 0.096 Mbp, includes the single conserved VirB4-like gene ("T4" in Figure 1). This stretch of 40 ORFs encodes thirteen proteins with signal peptides, four of which are *Frankia-*specific, as well as an additional nine *Frankia-*specific proteins (without signal peptides). The secreted proteins include the secretory lipase, the poly-gamma-glutamate biosynthesis protein, the S1/S6/Hap family peptidase, and a PHB depolymerase. In another region, between 0.87 and 0.92 Mbp, the conserved patatin gene is situated several genes downstream of the *secA *gene, and a few genes upstream of an operon of T2S genes. Downstream of the T2S genes is a conserved hypothetical protein, "Francci3_0789," which has Tat signal peptides predicted in all three strains. This region has five *Frankia-*specific secreted proteins (out of 40 ORFs), as well as an additional twelve signal peptide-containing proteins and nine *Frankia-*specific (non-secretory) proteins. Other *Frankia-*specific hypothetical proteins are clustered in regions near 4.1 Mbp and 4.7 Mbp (Figure 1). Several potential Tat-translocated proteins are found near 4.1 Mbp, and three highly conserved hypothetical proteins (Francci3_3886, Francci3_3889, and Francci3_3891) near 4.7 Mbp are located upstream of a channel protein of the hemolysin III family, which could facilitate protein export. The two peptidylprolyl isomerase genes are located near secretion machinery genes. The cyclophilin-type peptidylprolyl isomerase is located shortly downstream of the operon containing *yajC, secD*, and *secF*, while the other (FKBP-type) is found four genes away from the *tatA/tatC *genes. Two genes encoding proteins with putative Tat signal peptides predicted by TATFIND are roughly fifteen genes away from the *tatA/C *genes. One of these is a phosphoesterase; the other is annotated as a Dyp-type peroxidase (Additional File 3). Both of these types of proteins were shown to be secreted via the Tat pathway in other bacteria [43,44]. By situating genes in putative secretory regions of the genome, the gene neighborhoods in Figure 1 both highlight regions of interest and support the signal peptide predictions for these sequences.

## Conclusion

We screened three *Frankia *genomes for genes encoding protein secretion machinery and proteins with signal peptides. The protein secretion systems present in *Frankia *correlate with those found in other actinobacteria, but the predicted secretomes are reduced in size compared to those of other soil bacteria. We propose that this is an adaptation to an endosymbiotic lifestyle, in which *Frankia *secretes few proteins that might trigger host defenses. The *Frankia *genomes we examined do not have a conserved set of obvious polysaccharide-hydrolyzing enzymes. This finding challenges the hypothesis that *Frankia *hydrolyzes plant cell wall polymers during nodulation, though it is possible that the general glycosidases, esterases, or proteases contribute to this function. Genomic evidence suggests that polysaccharases are used to a greater or lesser degree depending on the lifestyle and mode of infection of particular strains, with strain CcI3 having lost secretory hydrolase genes present in the other two strains. The EAN genome also includes four arabinofuranosidases and two rhamnosidases without signal peptides, absent from CcI3 and ACN, which may be used to break down oligosacccharides derived from hemicelluloses. In the absence of plant cell wall degradation, it is likely that *Frankia *secretes novel classes of effector proteins to communicate with its host, a situation similar to that recently described in the biotrophic plant pathogenic fungus, *Ustilago maydis*, that is also deficient in cell wall hydrolytic enzymes[45]. The core *Frankia *secretome represents a conserved set of candidate proteins, including those with hydrolytic, surface-associated, solute-binding, and unknown functions, to assess for involvement in root infection. Mapping the locations of conserved secreted protein genes allowed us to identify genomic "hotspots" containing potentially secreted proteins and other unique frankial proteins. This genome-based *Frankia *secretome, combined with ongoing proteomics studies, will help navigate the way to a more complete understanding of the actinorhizal symbiosis.

## Methods

### Sequence analysis

The FASTA amino acid sequences of the three *Frankia *genomes [CcI3, NCBI RefSeq: NC_007777; ACN14a, NCBI RefSeq: NC_008278; EaN1pec, NCBI RefSeq: NC_009921] and from the *Streptomyces avermitilis *[GenBank: BA000030]*, Nocardia farcinica *[GenBank: AP006618]*, Mycobacterium tuberculosis *CDC1551 [GenBank: AE000516], *Kineococcus radiotolerans *[GenBank: CP000750], *Acidothermus cellulolyticus *[GenBank: CP00481], *Bradyrhizobium japonicum *[GenBank: BA000040], and *Sinorhizobium meliloti *[GenBank: AL591688] genomes (including the two *S. meliloti *plasmids, pSymA [GenBank: AE006469] and pSymB [GenBank: AL591985]) were obtained from the GenBank or NCBI RefSeq FTP sites [46,47]. All sequences were truncated to the first 70 amino acids and analyzed with the SignalP 3.0 program [20]. Sequences predicted to contain a signal peptide by SignalP were analyzed with TMHMM 2.0 [48] to determine the number of transmembrane (TM) domains. The CDS translations of the three *Frankia *genomes were also searched for the twin-arginine translocation (Tat) sequence motif using TATFIND 1.4 [21]. TATFIND searches for the motif [HAPKRNTGSDQE] **RR **[APKRNTGSDQE] [IWFLVYMCHAPNT] [ILVMF] within the first 35 amino acids of a sequence (where any of the amino acids in brackets satisfy the match) and scores positive if this motif is followed by hydrophobic stretch of at least 13 amino acids (within 22 downstream residues).

### Identification of hydrolytic enzymes

Hydrolytic enzymes were identified with the annotation provided by the Joint Genome Institute (CcI3 and EAN) or MaGe (ACN) [29,30]. To confirm that none of the signal peptide-containing hypothetical proteins were hydrolases, these sequences were searched for conserved domains with the SMART (Simple Modular Architecture Research Tool) program, and analyzed for hits to SCOP (Structural Classification of Proteins) protein superfamilies using the Superfamily 1.69 server [49,50]. The SCOP superfamily analysis led to the identification of four hypothetical proteins with slight similarity (E values between 10^-5 ^and 10^-22^) to the "(Trans)glycosidase" superfamily (see Table 2 and Figure 3E) and three with hits (E values between 10^-8 ^and 10^-27^) to the "Pectin lyase-like" superfamily (see Additional File 2). In addition, to confirm that the gene annotation and BLAST searches had not missed cellulase, pectinase, or xylanase genes in the frankial genomes, a PSI-BLAST search was performed [51]. The three genomes were searched using point-specific scoring matrices (PSSMs) of nine conserved hydrolase domains (three Pfam or COG domains for each type of hydrolase listed above) obtained from the NCBI conserved domain database (CDD) FTP site [52]. Only the previously identified polysaccharide-degrading enzymes were retrieved using this method.

For construction of phylogenetic trees, a representative subset of top-scoring BLAST hits was chosen and amino acid sequences were aligned with MUSCLE [53]. To generate trees, PhyML (maximum likelihood method) was used with the JTT substitution matrix and 100 non-parametric bootstrap replicates [54]. Trees were visualized using TreeView [55].

### Identification of the core secretome

To find orthologous proteins shared among the three strains, the complete CDSs from each *Frankia *genome were BLAST searched against those of the other two strains, using an E-value threshold of 10^-20 ^[56]. Using a custom Perl script, we identified the sequences that retrieved each other as the top BLAST hit (orthologs) in all three bi-directional comparisons. The amino acid sequences of these 6240 proteins (2080 from each strain) were then analyzed with SignalP 3.0 and TMHMM 2.0. Of these orthologous sequences, those with signal peptides predicted by one or both SignalP methods (NN or HMM) and having 0–2 transmembrane domains, in two or three of the strains, were considered to belong to the core *Frankia *secretome.

### Manual inspection of start codons

In cases where sequences from two strains had signal peptides predicted but the orthologous sequence from the third did not, the start codon region of that sequence was inspected manually, based on the amino acid sequence alignment with its orthologs. Alignments were viewed with the BLink tool from NCBI [57], and upstream regions were viewed with Artemis through the *Frankia alni *ACN14a Genoscope website, and the Integrated Microbial Genomes (IMG) website [29,58]. Depending on the conserved start position seen in the N-terminal sequence alignments, and on the presence of a putative Shine-Dalgarno sequence upstream of the start codon, some start sites were manually adjusted and analyzed again with SignalP. A total of 48 sequences in strain ACN were inspected (because CcI3 and EAN had signal peptides in the orthologous sequences); of these, 20 were predicted to contain a signal peptide after manual adjustment. Manual inspection of start codons was also carried out for sequences in the core proteome with a signal peptide predicted in only one strain (by both SignalP methods). Of 104 proteins inspected, only one additional signal peptide was predicted (ACN, gi|111220881). As a control measure, a subset of hydrolases lacking signal peptides was inspected to verify that signal peptides were not missed due to incorrect start codon annotation. After scanning the genome for intracellular hydrolases (of the types listed in Table 2, but not already present in this table), 96 were found in CcI3, 129 in ACN, and 195 in EAN. These were BLAST searched against each of the other genomes, and subgroups were selected in which the start position of the alignment between the query and subject sequences was > 10 amino acids apart. The N-terminal regions of 25 conserved intracellular hydrolases, and 8 hydrolases unique to EAN were inspected, and none of these resulted in new signal peptide predictions.

## Authors' contributions

JEM and DRB developed the study, performed the bioinformatics analyses, and wrote the manuscript. LST and PN provided the EaN1pec genome and the ACN14a genomes, respectively, and contributed to the editing of the manuscript.

## Supplementary Material

Additional File 1 **Secretion machinery genes**. This table lists the genes encoding secretion machinery components in the three *Frankia *genomes. Click here for file

Additional File 2 **Individual *Frankia *secretomes**. This table lists the proteins in each *Frankia *genome predicted to contain a signal peptide by both SignalP methods (NN and HMM), and with 0–2 trans-membrane domains predicted by TMHMM. Click here for file

Additional File 3 **Tat signal peptides predicted by TATFIND 1.4**. This table lists the proteins in each strain predicted to contain a Tat signal peptide by TATFIND 1.4, and shows their N-terminal sequences, including putative Tat motifs. Click here for file

Additional File 4 **The core *Frankia *secretome**. This table lists the 161 proteins in the core *Frankia *secretome, with SignalP predictions listed for each strain. Click here for file
